# Liver Sinusoidal Endothelial Cells in the Regulation of Immune Responses and Fibrosis in Metabolic Dysfunction-Associated Fatty Liver Disease

**DOI:** 10.3390/ijms26093988

**Published:** 2025-04-23

**Authors:** Munish Puri, Snehal Sonawane

**Affiliations:** 1 Onco-Immunology, Magnit Global, Folsom, CA 95630, USA; 2 Department of Pathology, University of Illinois, Chicago, IL 60612, USA; snehal@uic.edu

**Keywords:** LSECs, immune regulation, liver fibrosis, MAFLD liver disease, MASH, immune tolerance, LSEC dysfunction, therapeutic targets

## Abstract

Liver Sinusoidal Endothelial Cells (LSECs) play a crucial role in maintaining liver homeostasis, regulating immune responses, and fibrosis in liver diseases. This review explores the unique functions of LSECs in liver pathology, particularly their roles in immune tolerance, antigen presentation, and the modulation of hepatic stellate cells (HSCs) during fibrosis. LSECs act as key regulators of immune balance in the liver by preventing excessive immune activation while also filtering antigens and interacting with immune cells, including Kupffer cells and T cells. Metabolic Dysfunction-Associated Fatty Liver Disease(MAFLD) is significant because it can lead to advanced liver dysfunction, such as cirrhosis and liver cancer. The prevalence of Metabolic Associated Steatohepatitis (MASH) is increasing globally, particularly in the United States, and is closely linked to rising rates of obesity and type 2 diabetes. Early diagnosis and intervention are vital to prevent severe outcomes, highlighting the importance of studying LSECs in liver disease. However, during chronic liver diseases, LSECs undergo dysfunction, leading to their capillarization, loss of fenestrations, and promotion of pro-fibrotic signaling pathways such as Transforming growth factor-beta (TGF-β), which subsequently activates HSCs and contributes to the progression of liver fibrosis. The review also discusses the dynamic interaction between LSECs, HSCs, and other hepatic cells during the progression of liver diseases, emphasizing how changes in LSEC phenotype contribute to liver scarring and fibrosis. Furthermore, it highlights the potential of LSECs as therapeutic targets for modulating immune responses and preventing fibrosis in liver diseases. By restoring LSECs’ function and targeting pathways associated with their dysfunction, novel therapies could be developed to halt or reverse liver disease progression. The findings of this review reinforce the importance of LSECs in liver pathology and suggest that they hold significant promises as targets for future treatment strategies aimed at addressing chronic liver diseases.

## 1. Introduction

### Liver’s Unique Role in Immune Regulation and the Critical Function of LSECs

The liver plays a central role in immune regulation and functions to filter blood from the gastrointestinal tract, which is rich in antigens, nutrients, and toxins. Unlike other organs, the liver has developed mechanisms to maintain immune tolerance while simultaneously preventing infections and excessive immune responses. This immunological balance is critical for preventing damage to the liver itself, which can otherwise lead to inflammation and disease [1]. LSECs are structurally positioned next to the hepatic sinusoids and play a crucial role in this process [2]. Unlike typical endothelial cells, LSECs are highly specialized in their function of filtering blood, clearing pathogens, and presenting antigens. They regulate immune responses by interacting directly with immune cells such as T cells and Kupffer cells (resident macrophages), promoting tolerance to harmless antigens while allowing an appropriate immune response to harmful pathogens [3]. LSECs are, therefore, integral to maintaining hepatic homeostasis and preventing immune-mediated damage in the liver [4].

The prevalence of MASH is increasing globally, with a notable rise in the United States. It is estimated that by 2030, around 27 million adults in the US will be living with MAFLD [5]. This rise is closely linked to the increasing rates of obesity and type 2 diabetes, which are major risk factors for the development of Metabolic dysfunction-associated steatotic liver disease (MASLD) [6]. Clinically, MASH is challenging to diagnose due to the lack of specific markers for early liver fibrosis, which often leads to underdiagnosis. The progression of MAFLD can result in significant liver damage, necessitating early detection and intervention to prevent severe outcomes. Understanding the prevalence, risk factors, and clinical significance of MAFLD is crucial for developing effective public health strategies and therapeutic interventions to manage and mitigate this growing health concern [7]. MAFLD is broadly used for dysfunctional liver conditions characterized by fat accumulation due to metabolic issues, while MASH is a more severe form of MAFLD that includes inflammation and liver cell damage, including LSECs, potentially leading to fibrosis, cirrhosis, and Hepatocellular carcinoma (HCC).

LSECs are a type of endothelial cells that form the unique lining of the hepatic sinusoids. In addition to their filtering function, LSECs are central to maintaining immune homeostasis within the liver. TGF-β plays a dual role in liver pathology, acting as both a profibrotic and anti-inflammatory cytokine. In normal LSECs, TGF-β expression supports immune homeostasis. However, during chronic liver injury, its upregulation in activated LSECs drives hepatic stellate cell activation and extracellular matrix deposition, contributing to fibrosis progression. These cells are highly active in antigen presentation, taking up antigens from the blood and presenting them to immune cells, including T cells, in a manner that typically promotes immune tolerance rather than activation. This immune-regulatory function of LSECs is crucial, given the liver’s constant exposure to dietary and bacterial antigens from the gut [8]. By promoting tolerance, LSECs prevent unnecessary immune responses that could damage the liver, making them vital to both hepatic immune surveillance and the maintenance of systemic immune balance.

Advanced MAFLD progresses to liver fibrosis, characterized by excessive deposition of extracellular matrix proteins, which is a key feature of chronic liver diseases and a precursor to more severe conditions like cirrhosis and liver failure. In fibrosis, the liver’s standard architecture is progressively replaced by scar tissue, leading to impaired liver function [9]. This process is primarily driven by HSCs, which are activated in response to liver injury. LSECs play a crucial role in this process by interacting with HSCs and immune cells. In a healthy liver, LSECs maintain a quiescent state of HSCs through the release of regulatory molecules [10]. However, during liver injury or chronic inflammation, LSECs undergo phenotypic changes that promote the activation of HSCs, leading to fibrosis [11]. As fibrosis progresses, it compromises the liver’s regenerative capacity and can ultimately result in end-stage liver disease [12]. Understanding LSECs’ contributions to immune regulation and fibrosis is crucial for developing therapeutic strategies to reduce liver damage and improve patient outcomes [13,14].

The primary objective of this review is to examine the role of LSECs in the regulation of immune responses and the development of fibrosis in chronic liver diseases. While the liver’s immune environment has been extensively studied, there is a growing recognition of the unique contributions of LSECs to liver pathology. Despite their critical roles in immune tolerance, pathogen clearance, and fibrosis modulation, LSECs have historically been underexplored compared to other hepatic cells such as hepatocytes and Kupffer cells. A deeper understanding of LSECs is crucial for developing novel therapeutic approaches that target these cells to prevent or reverse fibrosis and regulate immune responses [15]. By synthesizing recent advances in this area, this review aims to highlight the potential of LSECs as both diagnostic biomarkers and therapeutic targets in the treatment of liver diseases, addressing gaps in the current literature and guiding future research efforts [16]. Finally, it will provide insights into emerging research areas and potential future studies on LSECs, summarizing key findings and the importance of LSECs in liver pathology.

## 2. Liver Sinusoidal Endothelial Cells: Structure and Function

### 2.1. Anatomy and Physiology of LSECs

**Figure 1:**
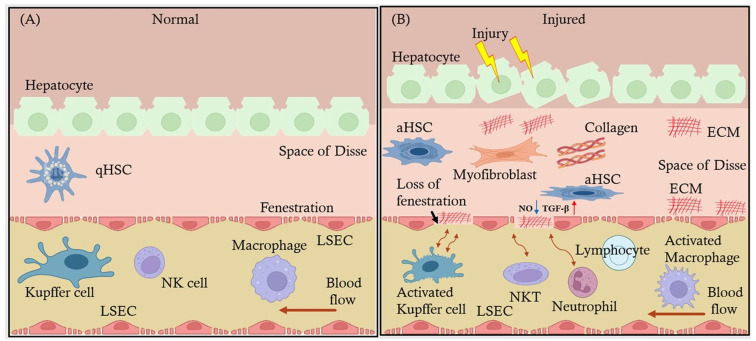
Structural and functional changes in liver LSECs in normal and injured liver. This image illustrates the structural and functional differences in LSECs between a normal liver (**A**) and an injured liver (**B**). In the normal liver, hepatocytes are aligned with fenestrated LSECs, allowing for efficient blood flow and interaction with Kupffer cells, macrophages, and NK Cells. Quiscent (q)HSCs are present in the space of Disse. In contrast, the injured liver shows hepatocyte damage, loss of fenestration in LSECs, and the activation of HSCs (aHSCs) into myofibroblasts, leading to extracellular matrix (ECM) deposition and collagen accumulation. This results in the activation of Kupffer cells, macrophages, and the presence of lymphocytes, natural killer cells (NKTs), and neutrophils, indicating an inflammatory response. The figure also highlights the molecular changes in LSECs, such as increased TGF-β expression (red up arrow) and decreased nitric oxide (NO) production (down blue arrow), and their interactions with other hepatic cells during liver injury. This figure also depicts ECM thickening in the space of Disse during fibrosis.

LSECs’ fenestrations filter blood efficiently, controlling the passage of lipoproteins, proteins, and immune molecules into the liver parenchyma [17]. There is variation in the size and number of fenestrae based on their location. The periportal LSECs have larger fenestrae and are fewer in number, while pericentral LSECs have smaller and numerous fenestrae [18]. Positioned between the blood flow and hepatocytes, LSECs play a crucial role in the clearance of pathogens, toxins, and waste products from the blood, such as metabolic byproducts and cellular debris. These waste products include substances like bilirubin, ammonia, and other metabolic end products that need to be efficiently removed to maintain liver and overall body health. Through scavenger receptors, LSECs rapidly remove circulating antigens, immune complexes, and apoptotic cells, contributing to the liver’s role as a detoxification organ [7]. Figure 1 illustrates the structure and function of LSECs in the normal and injured stages. The image highlights the critical role of LSECs in maintaining liver function and the pathological changes that occur during liver injury.

In addition to their filtering function, LSECs are central to maintaining immune homeostasis within the liver. These cells are highly active in antigen presentation, taking up antigens from the blood and presenting them to immune cells, including T cells, in a manner that typically promotes immune tolerance rather than activation [19]. This immune-regulatory function of LSECs is crucial, given the liver’s constant exposure to dietary and bacterial antigens from the gut. In fibrosis following steatohepatitis, ECM is deposited in the space of Disse, which eventually surrounds hepatocytes and thickens the space of Disse, as illustrated in Figure 1 [20]. By promoting tolerance, LSECs prevent unnecessary immune responses that could damage the liver, making them vital to both hepatic immune surveillance and the maintenance of systemic immune balance [21].

Capillarization refers to the transformation of LSECs into a phenotype resembling continuous capillaries, whereas de-capillarization represents the reversal of capillarization, where LSECs regain their fenestrations and lose the basement membrane. The capillarization process involves not only the loss of fenestrations but also a reduction in tight gap junctions between cells, leading to increased barrier integrity and altered functionality, further resembling microvascular endothelial cells. Electron microscopic studies have also revealed that there are some structural differences between pericentral and periportal LSECs and they also carry different molecular signature and functions. While the periportal LSECs are mainly involved in nutrient uptake, cholesterol synthesis, and lipid oxidation, the pericentral LSECs are mainly involved in glycolysis and xenobiotic metabolism [22].

### 2.2. Interaction of LSECs with Other Hepatic Cells: Kupffer Cells, HSCs, and Hepatocytes

LSECs do not function in isolation; their interaction with other hepatic cells is central to liver health and disease. One of the most essential cellular partners of LSECs is Kupffer cells, the resident macrophages of the liver. Kupffer cells are located within the liver sinusoids and are involved in immune surveillance and phagocytosis. LSECs and Kupffer cells collaborate in maintaining immune tolerance; for instance, LSECs uptake and present antigens to Kupffer cells, which then help suppress excessive immune responses. Kupffer cells also respond to inflammatory signals from LSECs, orchestrating broader immune responses when necessary, such as during infections or liver injury [23,24].

LSECs also play a key role in the activation and regulation of HSCs, the primary cells responsible for liver fibrosis. Under normal conditions, LSECs help maintain HSCs in a quiescent state by releasing soluble factors like nitric oxide, which suppress HSC activation [25]. However, in the context of liver injury or chronic inflammation, LSECs undergo changes that promote HSC activation. These changes include an altered secretion profile, producing increased levels of pro-fibrotic cytokines like TGF-β, and higher expression of adhesion molecules such as VCAM1, promoting the recruitment of inflammatory cells to the liver. Activated HSCs transform into myofibroblasts, which produce collagen and extracellular matrix proteins, leading to fibrosis. This crosstalk between LSECs and HSCs is a critical factor in liver disease progression, especially in chronic liver conditions like MAFLD [26].

LSECs also interact with hepatocytes, the primary functional cells of the liver. Hepatocytes depend on LSECs for the efficient delivery of nutrients and signaling molecules. The fenestrations in LSECs allow the transfer of essential molecules such as lipoproteins and hormones to hepatocytes, while hepatocytes, in return, help maintain LSECs’ function by releasing regulatory factors [27]. During liver injury, the interaction between LSECs and hepatocytes becomes significant, as LSECs respond to hepatocyte damage by initiating immune responses and modulating fibrosis through their interaction with HSCs [28]. In conditions like cirrhosis, these interactions become dysregulated, contributing to liver dysfunction and disease progression [29].

LSECs are central to several critical liver functions and processes. Their interactions with Kupffer cells, HSCs, and hepatocytes not only regulate immune responses but also maintain overall liver homeostasis. Understanding these interactions is crucial for identifying new therapeutic approaches aimed at treating liver diseases by targeting the LSEC-mediated pathways. LSECs play a crucial role in liver regeneration and post-transplant status. During liver regeneration, LSECs secrete growth factors such as VEGF and HGF, which promote hepatocyte proliferation and tissue repair. In the context of liver transplantation, LSECs help maintain immune tolerance and prevent graft rejection by inducing regulatory T cells (Tregs) and suppressing effector T cells. Enhancing the immunomodulatory functions of LSECs could improve graft survival and reduce the need for long-term immunosuppression in transplant recipients.

Human studies have demonstrated that LSECs capillarization and de-capillarization play significant roles in liver fibrosis regression and progression. Capillarization contributes to the progression of liver fibrosis by impairing LSECs’ function and promoting HSCs activation. Conversely, de-capillarization, the restoration of fenestrations, is associated with fibrosis regression and improved liver function. Studies have shown that targeting LSECs capillarization can be a potential therapeutic strategy for reversing liver fibrosis and promoting liver regeneration [30,31].

## 3. LSECs in Immune Regulation

Unlike typical endothelial cells, LSECs act as key regulators of hepatic microcirculation and immune surveillance. LSECs play a crucial role in modulating the immune response by presenting antigens and promoting immune tolerance in the liver’s unique immune environment, which is constantly exposed to gut-derived antigens and circulating pathogens. This delicate balance ensures that the liver can prevent inappropriate immune activation while still mounting effective immune responses when necessary [32].

### 3.1. Antigen Presentation and Immune Tolerance

LSECs are involved in the presentation of antigens to immune cells, especially T cells, a process critical for maintaining immune tolerance. Unlike professional antigen-presenting cells (APCs), such as dendritic cells, LSECs have a unique capability to induce tolerance rather than immune activation. They do this by presenting antigens via major histocompatibility complex (MHC) class I and class II molecules to naive T cells, leading to the induction of CD8⁺ T cell tolerance through mechanisms like clonal deletion and anergy [33].

Moreover, LSECs express low levels of costimulatory molecules, such as CD80 and CD86, which are essential for full T cell activation. This deficiency in costimulation skews T cell responses towards tolerance rather than effector functions. Additionally, LSECs secrete immunomodulatory molecules like interleukin-10 (IL-10) and transforming growth factor-beta (TGF-β), which further support the development of Tregs and dampen pro-inflammatory T cell responses [34].

### 3.2. LSECs and Immune Cell Communication

LSECs communicate with various immune cells, including T cells and Kupffer cells, to regulate immune responses and prevent excessive immune activation.

#### 3.2.1. Interaction with T Cells

As previously discussed, liver diseases progress through a continuum, starting from early inflammation and advancing to fibrosis. The cross-presentation of exogenous antigens by liver sinusoidal endothelial cells (LSECs) to CD8^+^ T cells, in the absence of sufficient costimulatory signals, triggers T cell apoptosis or induces a non-responsive (anergic) state, thereby preventing cytotoxic T lymphocyte (CTL) activation and subsequent tissue damage [35].

#### 3.2.2. Interaction with Kupffer Cells

Kupffer cells play a crucial role in maintaining immune homeostasis in the liver. LSECs closely interact with Kupffer cells and other liver macrophages to modulate immune responses. Through the release of anti-inflammatory cytokines, LSECs contribute to maintaining the tolerogenic microenvironment of the liver. In turn, Kupffer cells provide feedback to LSECs by producing signaling molecules like IL-10 and TGF-β, which further enhance the immunosuppressive functions of LSECs [36].

The crosstalk between LSECs and Kupffer cells is essential for preventing excessive immune activation in the liver. Dysregulation of this communication can lead to increased inflammation, as Kupffer cells can switch to a pro-inflammatory phenotype under certain conditions, such as chronic liver disease or infections.

### 3.3. Role of LSECs in Liver Diseases

LSECs play crucial roles, such as promoting inflammation and fibrosis in advanced MAFLD and the development of portal hypertension and liver dysfunction in cirrhosis.

#### 3.3.1. Critical Role of LSECs in MAFLD

In MAFLD, the liver undergoes a series of progressive changes starting from hepatic steatosis (fatty liver) to more advanced stages. Initially, hepatic steatosis is characterized by the accumulation of excess fat within hepatocytes. As the disease progresses, some patients develop MASH, which is marked by inflammation and hepatocyte injury in addition to fat accumulation. This stage is critical as it signifies a transition from simple steatosis to a more severe form of liver disease. As MAFLD advances to MASH, LSECs undergo morphological changes, including capillarization and loss of fenestrations. These changes impair their ability to regulate immune tolerance, leading to increased infiltration of pro-inflammatory immune cells such as CD8⁺ T cells and macrophages. The resulting inflammation exacerbates liver damage, promoting fibrosis and the progression of advanced MASH to cirrhosis.

Understanding the role of LSECs in the progression from MAFLD to MASH is crucial for developing therapeutic strategies aimed at preventing or reversing liver damage. By targeting the pathways involved in LSECs dysfunction, it may be possible to mitigate inflammation, reduce fibrosis, and improve liver health.

#### 3.3.2. Role of LSECs in Cirrhosis

During liver fibrosis, LSECs start behaving more like typical vascular endothelial cells and reduce their ability to clear antigens and maintain immune tolerance. The dysfunction of LSECs contributes to a pro-inflammatory environment in the liver, with enhanced recruitment of immune cells like monocytes and neutrophils. Over time, this chronic inflammation leads to excessive deposition of ECM by aHSC, driving fibrosis and eventually cirrhosis [37]. LSECs are critical for maintaining immune tolerance in the liver through antigen presentation, communication with immune cells, and the release of anti-inflammatory cytokines. Understanding the mechanisms by which LSECs regulate immune responses and how their dysfunction contributes to liver pathology may provide new therapeutic opportunities for inflammatory liver diseases.

## 4. LSECs in Liver Fibrosis

### 4.1. LSECs’ Influence on Hepatic Stellate Cell Activation and Collagen Deposition Leading to Fibrosis

LSECs play a critical role in the regulation of HSCs, which are the primary cells responsible for collagen deposition and fibrosis in the liver. Under healthy conditions, LSECs help maintain HSCs in a quiescent, non-fibrogenic state by producing soluble factors such as nitric oxide (NO) [38]. NO helps to keep HSCs inactive and prevents the excessive production of ECM proteins, including collagen. However, in the context of chronic liver injury, whether due to alcohol abuse or MAFLD, LSECs undergo functional changes that lead to the activation of HSCs [39].

When LSECs become dysfunctional, they lose their ability to produce sufficient NO and other antifibrotic signals. As a result, HSCs are activated, transforming into myofibroblasts—cells responsible for secreting large amounts of collagen and other ECM proteins. This excessive collagen deposition disrupts the liver’s typical architecture, leading to scarring and contributing to fibrosis progression [40]. Additionally, capillarization of LSECs exacerbates fibrosis by restricting nutrient and oxygen supply to hepatocytes, thereby promoting liver damage and inflammation [41].

### 4.2. Signaling Pathways Involved in LSEC-Mediated Fibrosis and Endothelial Dysfunction

Several signaling pathways are involved in LSEC-mediated fibrosis and endothelial dysfunction. One of the most crucial pathways is the TGF-β signaling pathway. TGF-β is a potent pro-fibrotic cytokine that plays a significant role in the activation of HSCs. Dysfunctional LSECs can produce increased levels of TGF-β, which directly stimulates HSC activation and collagen production. Furthermore, LSECs themselves become more susceptible to the effects of TGF-β, which contributes to their capillarization and loss of normal function [42].

Another significant pathway is the vascular endothelial growth factor (VEGF) signaling pathway. Under normal conditions, VEGF maintains the fenestrated structure of LSECs and supports their survival. In chronic liver disease, reduced VEGF signaling leads to capillarization of LSECs, contributing to fibrosis. Capillarized LSECs exhibit characteristics similar to vascular endothelial cells, further promoting inflammation and fibrosis by interacting with immune cells and HSCs pathologically [43].

Oxidative stress is also a significant factor in LSEC-mediated fibrosis. In conditions such as alcoholic liver disease or fatty liver disease, increased oxidative stress leads to the production of reactive oxygen species (ROS), which damage LSECs and contribute to their dysfunction. This, in turn, leads to the activation of pro-fibrotic signaling pathways, such as platelet-derived growth factor (PDGF), which further stimulates HSC activation and collagen production [44].

### 4.3. Changes in LSECs’ Phenotype Exacerbate Liver Scarring

Studies show that changes in LSECs’ phenotype, such as capillarization and loss of anti-fibrotic signaling, significantly exacerbate fibrosis and liver scarring. LSECs from fibrotic livers exhibited increased expression of TGF-β and decreased expression of NO-producing enzymes. These changes were correlated with heightened HSC activation and collagen deposition, indicating a direct role of LSEC dysfunction in fibrosis progression [45]. Restoring NO levels in LSECs helped mitigate fibrosis by inhibiting HSC activation.

Similarly, other studies emphasized that capillarized LSECs were less effective at filtering blood and regulating immune cell recruitment. The loss of fenestrations in LSECs led to the enhanced recruitment of inflammatory macrophages, which further stimulated HSC activation and promoted liver scarring. Targeting LSEC capillarization was proposed as a potential therapeutic strategy to prevent fibrosis [46].

These findings underscore the critical role of LSECs in the progression of liver fibrosis and suggest that therapeutic approaches aimed at restoring LSEC function could be effective in reducing fibrosis and preventing liver disease progression.

### 4.4. LSECs in Liver Disease Progression

In MAFLD, LSECs contribute to disease progression by becoming dysfunctional as fat accumulation increases. As MAFLD progresses to MASH, LSECs experience capillarization and take on characteristics like vascular endothelial cells. LSECs fail to regulate immune responses, scavenging functions, and portal pressure, promoting fibrosis and inflammation. Dysfunctional LSECs also produce inflammatory cytokines like tumor necrosis factor-alpha (TNF-α) and interleukin-6 (IL-6), which amplify local inflammation and exacerbate liver injury [47]. These inflammatory signals further promote the activation of HSCs, leading to increased collagen deposition and fibrosis [48].

### 4.5. Interplay Between LSECs and Other Liver Cells During Liver Disease Progression

As previously discussed, the progression of liver diseases from early inflammation to advance fibrosis and cirrhosis involves a dynamic relationship between LSECs and other liver cells including hepatocytes, HSCs, and immune cells like Kupffer cells. This cellular crosstalk is critical in determining the outcome of liver injury and the rate at which chronic liver diseases progress.

In the early stages of MAFLD, LSECs play a protective role by maintaining immune tolerance and preventing excessive immune activation. LSECs filter antigens from the bloodstream and communicate with Kupffer cells to modulate immune responses. However, as liver injury persists, LSECs lose their protective role. Inflammatory signals from damaged hepatocytes and infiltrating immune cells, such as macrophages and neutrophils, trigger LSECs to release pro-inflammatory and pro-fibrotic factors [20].

As inflammation becomes chronic, LSECs begin to interact with HSCs more pathologically. Normally, LSECs release antifibrotic signals such as NO, which keep HSCs in a quiescent state. However, during liver injury, LSECs reduce NO production and increase the release of TGF-β and PDGF. These signals activate HSCs, causing them to differentiate into myofibroblasts, which produce collagen and other extracellular matrix proteins that lead to fibrosis. This activation of HSCs is a hallmark of the fibrotic response in chronic liver diseases.

In advanced fibrosis and cirrhosis, the liver architecture becomes severely disrupted. LSECs promote further fibrosis by supporting angiogenesis, which is mediated by VEGF. This abnormal blood vessel formation creates a hypoxic environment that perpetuates liver scarring and dysfunction. At this stage, the interplay between LSECs, HSCs, and immune cells becomes a vicious cycle with continuous collagen deposition, inflammation, and liver remodeling. Cirrhotic livers also show increased interaction between LSECs, and macrophages recruited from the bloodstream, which produce additional pro-inflammatory and pro-fibrotic signals, accelerating liver failure.

LSECs are central to both the initiation and progression of liver diseases. They play a dual role in both protecting and damaging the liver, depending on the severity or degree of the disease. Understanding the mechanisms behind LSEC dysfunction and their interactions with other liver cells offers potential therapeutic targets to halt or reverse liver disease progression.

## 5. Stages of MAFLD Liver and Therapeutic Strategies

### 5.1. Healthy Liver

A healthy liver maintains its normal structure and function, with LSECs playing a crucial role in filtering blood, clearing pathogens, and maintaining immune tolerance.

### 5.2. Hepatic Steatosis (Fatty Liver)

Hepatic steatosis or fatty liver is the initial stage of MAFLD, characterized by the accumulation of excess fat within hepatocytes. This stage is often asymptomatic and can be reversible with lifestyle modifications such as diet and exercise. During this stage, LSECs maintain their fenestrated structure [49]. Therapeutic strategies at this stage focus on lifestyle interventions and metabolic regulation to prevent progression to more severe stages.

### 5.3. Steatohepatitis (MASH) (Fat + Inflammation)

As MAFLD progresses, some patients develop MASH, which is marked by inflammation and hepatocyte injury in addition to fat accumulation. This stage signifies a transition from simple steatosis to a more severe form of liver disease. LSECs undergo morphological changes, impairing their ability to regulate immune tolerance. This leads to increased infiltration of pro-inflammatory immune cells, exacerbating liver damage and promoting fibrosis. Therapeutic strategies at this stage focus on reducing inflammation and preventing further liver injury.

### 5.4. Advanced MASH (Fibrosis) (Fat + Inflammation + ECM)

Persistent inflammation and liver cell injury result in fibrosis, where scar tissue forms and disrupts the normal liver architecture. LSECs undergo significant capillarization, contributing to the progression of fibrosis by promoting the activation of HSCs and the deposition of ECM [50]. Therapeutic strategies at this stage focus on antifibrotic agents that target the ECM-LSECs interaction and mechano-transduction pathways, aiming to reverse fibrosis and restore normal liver function.

### 5.5. Cirrhosis

Advanced fibrosis leads to cirrhosis, characterized by extensive scarring that replaces healthy liver tissue. Cirrhosis severely impairs liver function and can lead to liver failure. LSECs are extensively capillarized, further exacerbating liver dysfunction and contributing to portal hypertension and impaired blood flow. Therapeutic strategies at this stage are more challenging and focus on managing symptoms, reducing portal hypertension, and improving liver regeneration. Potential therapies include agents that enhance endothelial function and reduce fibrosis [51].

### 5.6. Hepatocellular Carcinoma (HCC)

In some cases, cirrhosis can progress to HCC, a type of liver cancer. LSECs may contribute to the tumor microenvironment by promoting angiogenesis and providing a supportive niche for cancer cells. Therapeutic strategies at this stage focus on targeting the tumor vasculature and inhibiting angiogenesis, potentially using anti-angiogenic agents and immune modulators [52].

## 6. LSECs as Therapeutic Target

### 6.1. Therapeutic Approaches Targeting LSECs to Prevent or Reverse Liver Fibrosis and Modulate Immune Responses

**Figure 2:**
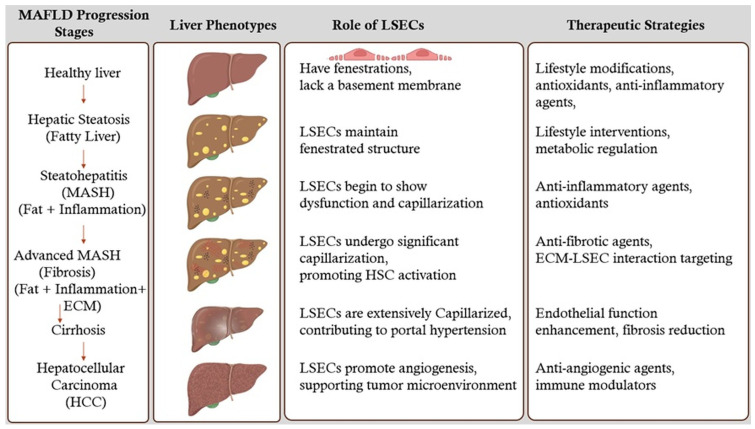
MAFLD progression stages and the role of LSECs in therapeutic strategies. This figure illustrates the progression stages of MAFLD with associated liver phenotypes and the role of LSECs. It highlights the importance of understanding LSECs in these stages to design and develop therapeutic strategies for identifying drug targets. The stages include Healthy Liver, Hepatic Steatosis (Fatty Liver), Steatohepatitis (MASH) (Fat + Inflammation), Advanced MASH (Fibrosis) (Fat + Inflammation + ECM), Cirrhosis, and Hepatocellular Carcinoma (HCC). Each stage is accompanied by visual representations of liver phenotypes, descriptions of LSECs’ roles, and suggested therapeutic strategies, emphasizing the critical role of LSECs in disease progression and potential drug targets.

Targeting LSECs has emerged as a promising therapeutic strategy to prevent or reverse liver fibrosis and modulate immune responses. Figure 2 illustrates the MAFLD liver progression stages, the role of LSECs, and therapeutic strategies. This section reviews the potential therapeutic approaches targeting LSECs and discusses current and emerging treatments aimed at enhancing LSECs’ function or inhibiting the fibrotic cascade.

### 6.2. Potential Therapeutic Approaches Targeting LSECs

#### 6.2.1. Restoration of LSECs’ Phenotype and Function

Under healthy conditions, LSECs exhibit fenestrations that facilitate the exchange of substrates between the blood and hepatocytes. Therapeutic strategies aimed at restoring the fenestrated phenotype and functional integrity of LSECs are thus essential, as illustrated in Table 1.

- Vasoactive Agents: Agents such as VEGF and NO donors can promote the maintenance of LSECs’ fenestrations and prevent capillarization. Enhancing VEGF signaling has been shown to sustain LSECs’ differentiation and function [17].
- Shear Stress Modulators: LSECs respond to shear stress induced by blood flow. Modulating shear stress through mechanical or pharmacological means can influence LSECs’ phenotype and prevent fibrosis progression [53].

#### 6.2.2. Inhibition of Pro-Fibrotic Signaling Pathways

LSECs interact closely with HSCs. Dysfunctional LSECs release factors that activate HSCs, promoting fibrosis. Targeting the signaling pathways involved in LSECs-mediated HSC activation is a viable therapeutic approach.

- TGF-β Signaling Inhibitors: TGF-β is a key cytokine involved in HSCs activation. Inhibiting TGF-β signaling in LSECs can reduce their pro-fibrotic influence on HSCs. TGF-β serves a dual role in the immune system and liver pathology. As an anti-inflammatory cytokine, it promotes immune tolerance by supporting regulatory T cell differentiation and suppressing pro-inflammatory responses. This mechanism is critical for maintaining immune homeostasis in normal LSECs and limiting inflammation-driven tissue damage during early stages of liver disease [54].
- Notch Pathway Modulators: The Notch signaling pathway in LSECs influences vascular remodeling and fibrogenesis. Modulating Notch signaling may attenuate fibrotic responses [55].

#### 6.2.3. Modulation of Immune Responses

LSECs are integral to hepatic immune surveillance and tolerance. In liver fibrosis, LSEC dysfunction contributes to an aberrant immune environment. Therapeutic strategies that restore proper immune modulation by LSECs can mitigate fibrosis and inflammation.

- Immune Checkpoint Modulators: targeting immune checkpoints such as PD-L1 on LSECs can regulate T cell responses, reducing chronic inflammation and fibrogenesis [56].
- Cytokine Therapy: administering anti-inflammatory cytokines or inhibitors of pro-inflammatory cytokines can rebalance the immune milieu toward fibrosis resolution [57].

### 6.3. Current and Emerging Treatments Targeting LSECs

#### 6.3.1. Pharmacological Agents Enhancing LSECs’ Function

Several drugs are currently under investigation or in use that aim to improve LSECs’ function, thereby preventing or reversing liver fibrosis.

- Statins: Beyond their lipid-lowering effects, statins have been shown to improve endothelial function. In LSECs, statins can enhance nitric oxide production, maintain fenestrations, and inhibit HSC activation. Clinical studies have suggested that statin therapy may slow fibrosis progression in chronic liver diseases [58].
- Angiogenesis Inhibitors: While angiogenesis is often associated with pathological conditions, controlled inhibition can prevent aberrant vascular remodeling in fibrosis. Agents targeting VEGF receptors may help maintain LSECs structure and function [59].
- FXR Agonists: Farnesoid X receptor (FXR) agonists, such as obeticholic acid, have hepatoprotective and anti-fibrotic effects. They modulate bile acid metabolism and exhibit anti-inflammatory properties that indirectly benefit LSECs’ function [60].

#### 6.3.2. Antifibrotic Therapies Targeting the Fibrotic Cascade

Emerging treatments focus on directly inhibiting the fibrotic cascade, with LSECs being a critical target for these interventions.

- Pirfenidone and Nintedanib: approved for idiopathic pulmonary fibrosis, these agents have shown potential in liver fibrosis by inhibiting fibrogenic pathways, including those mediated by LSECs [61].
- Galectin-3 Inhibitors: Galectin-3 is involved in fibrogenesis and inflammation. Inhibiting galectin-3 can reduce HSCs activation and ECM production, with beneficial effects on LSECs’ function [62]. More potent and specific inhibitors are currently being developed to enhance therapeutic outcomes [63].

#### 6.3.3. Regenerative and Cell-Based Therapies

Advancements in regenerative medicine offer innovative approaches to restore healthy LSECs populations and liver architecture.

- Stem Cell Therapy: Mesenchymal stem cells (MSCs) and endothelial progenitor cells (EPCs) can differentiate into functional LSECs, promoting vascular repair and reducing fibrosis [64].
- Gene Therapy: delivery of genes encoding protective factors such as VEGF or antifibrotic proteins to LSECs can enhance their regenerative capacity and inhibit fibrogenic signaling [65].

#### 6.3.4. Nanotechnology and Targeted Drug Delivery

Nanocarriers can be designed to deliver therapeutic agents specifically to LSECs, enhancing drug efficacy and minimizing off-target effects.

- LSEC-Targeted Nanoparticles: utilizing ligands that bind to receptors uniquely expressed on LSECs, such as mannose receptors, allows for precise delivery of antifibrotic drugs or siRNA molecules to these cells [66].
- Controlled Release Systems: nanotechnology-enabled systems can provide sustained release of therapeutic agents, ensuring prolonged LSECs modulation and fibrosis inhibition.

#### 6.3.5. Biomolecular Inhibitors and Small Molecules

Discovery of novel biomolecules and small molecules that specifically modulate LSECs’ function is a burgeoning field.

- MicroRNA Modulators: MicroRNAs (miRNAs) regulate gene expression in LSECs. Therapeutics that mimic or inhibit specific miRNAs can alter LSECs’ behavior to favor antifibrotic outcomes [67].
- Small Molecule Inhibitors: Identifying small molecules that inhibit profibrotic enzymes or signaling molecules. LSECs can provide targeted antifibrotic effects [68].

### 6.4. Clinical Implications

The therapeutic targeting of LSECs holds significant promise for the treatment of liver fibrosis. Current treatments, while beneficial, often exhibit limited efficacy or adverse effects. Emerging therapies, particularly those leveraging regenerative medicine and nanotechnology, offer novel mechanisms to modulate LSECs’ function and the hepatic microenvironment precisely. Research efforts in this direction should focus on the following:

- Biomarker Development: identifying reliable biomarkers for LSECs dysfunction can aid in patient stratification and monitoring therapeutic responses [69].
- Combination Therapies: combining LSEC-targeted therapies with other antifibrotic agents may produce synergistic effects, enhancing overall treatment efficacy [70].
- Personalized Medicine: tailoring therapies based on individual patient profiles and specific LSECs pathophysiology could optimize treatment outcomes.
- Clinical Trials: rigorous clinical testing of emerging therapies is essential to establish safety, efficacy, and optimal dosing strategies for patients with liver fibrosis [71].

LSECs are critical players in the pathogenesis and resolution of liver fibrosis. Therapeutic strategies that target LSECs to restore their normal function, inhibit pro-fibrotic signaling, and modulate immune responses represent a promising avenue for combating liver fibrosis. Ongoing drug development research and clinical advancements will likely expand the repertoire of effective LSEC-targeted therapies, ultimately improving outcomes for patients with chronic liver diseases. A study performed on developing biomarkers discusses the use of an automated machine learning (AutoML) diagnostic support system as a computational biomarker for detecting drug-induced liver injury (DILI) patterns in liver pathology. It highlights the effectiveness of the developed assay in accurately classifying necrotic injury patterns with high precision, providing a valuable tool for early detection and assessment of liver toxicity in drug development [72].

## 7. Current Challenges and Future Directions

### 7.1. Gaps in the Current Understanding of LSECs in Liver Disease

Despite significant advancements in understanding the role of LSECs in liver disease, there are still notable gaps that hinder the development of effective treatments targeting these cells. One of the primary challenges lies in the complexity of studying LSECs in the context of liver disease, mainly due to the limitations of in vivo models. For instance, rodent LSECs differ in some key physiological respects from human LSECs, particularly in their immune regulatory functions, making it challenging to model immune tolerance and disease progression accurately. Moreover, most existing models do not adequately replicate chronic liver conditions, such as MAFLD and cirrhosis, where long-term interactions between LSECs and other liver cells are crucial to disease progression.

Additionally, clinical studies investigating the specific roles of LSECs in liver disease are still limited. Most research on LSECs has been conducted in preclinical settings, and the number of human trials focused on LSECs as a therapeutic target is limited. While LSECs contribute significantly to liver fibrosis and immune regulation, clinical studies that explore LSECs’ direct involvement in human liver diseases are still lacking. The heterogeneity of LSECs in different stages of liver disease, including the transition from a healthy to a diseased state, is not fully understood. Further clinical research is necessary to elucidate how changes in LSECs’ phenotype and function contribute to disease outcomes and to identify potential biomarkers that can predict fibrosis progression or liver dysfunction.

### 7.2. LSECs Dysfunction in Liver Disease

LSECs play a pivotal role in maintaining liver homeostasis, and their dysfunction is a central theme in the progression of liver diseases. This dysfunction contributes to the activation of HSCs and the subsequent development of fibrosis. The loss of LSECs’ function exacerbates inflammation and promotes a pro-fibrotic environment, highlighting their critical role in liver pathology.

### 7.3. Unifying Hypothesis of LSECs-Mediated Pathology in MAFLD

Integrating findings across various sections of this review, we propose a unifying hypothesis that LSECs dysfunction is one of the key drivers of MAFLD progression. This dysfunction triggers a cascade of events, including increased inflammation, HSCs activation, and extracellular matrix deposition, ultimately resulting in fibrosis and cirrhosis. Understanding the mechanisms underlying LSECs dysfunction provides a comprehensive framework for developing targeted therapies to mitigate liver disease progression.

### 7.4. Emerging Research Areas

Emerging research areas such as organoids and computational modeling offer promising avenues for studying LSECs’ function. Organoids, which are three-dimensional cell culture systems that mimic the architecture and function of organs, can provide valuable insights into LSECs’ behavior in a controlled environment [73]. Computational modeling, on the other hand, allows for the simulation of complex biological processes, enabling researchers to predict the effects of LSECs dysfunction and identify potential therapeutic targets [74]. These innovative approaches hold the potential to advance our understanding of LSEC-mediated pathology and inform the development of novel treatments.

### 7.5. Future Research Directions: Role of LSECs in Liver Regeneration and Transplantation

There is a rising need to understand LSECs’ potential roles in liver regeneration and liver transplantation. LSECs have shown promise in promoting liver regeneration due to their ability to maintain immune tolerance and modulate inflammation. Research has suggested that LSECs contribute to hepatocyte proliferation by secreting growth factors such as VEGF and hepatocyte growth factor (HGF). However, the mechanisms by which LSECs promote liver regeneration, especially in a chronically damaged liver, remain poorly understood. More studies are required to focus on elucidating these mechanisms and exploring how LSECs can be manipulated to enhance liver regeneration following injury or surgical resection. Investigating LSECs’ regenerative potential could lead to novel strategies to treat liver diseases that currently require liver transplantation.

In the context of liver transplantation, LSECs play a critical role in maintaining immune tolerance and preventing graft rejection. One promising research direction is exploring how to enhance the immunomodulatory functions of LSECs to prevent or mitigate immune rejection in liver transplant patients. The ability of LSECs to induce Tregs and suppress effector T cells makes them an attractive target for therapies aimed at reducing the need for long-term immunosuppression in transplant recipients. Gene editing technologies, such as CRISPR-Cas9, could be utilized to modify LSECs in ways that enhance their ability to promote tolerance and protect against rejection. Furthermore, LSECs could be used in bioengineered liver grafts or as part of cell-based therapies to support graft survival and function.

While significant progress has been made in understanding LSECs, there remain considerable gaps, particularly in modeling and clinical research. The focus could be on developing more accurate models, investigating LSECs’ role in liver regeneration and transplantation, and exploring their therapeutic potential. By addressing these challenges, we can unlock new pathways to treat and potentially reverse liver diseases, improving patient outcomes.

## 8. Limitations

### 8.1. Hurdles in Studying LSECs

Studying LSECs presents several difficulties, including the limitations of in vivo and in vitro models. In vivo models, such as rodent models, often fail to fully replicate the human liver’s unique environment and cellular interactions [75]. Moreover, the progression of liver disease in humans spans decades, a time frame that short-term animal models cannot replicate. Additionally, the lack of specific biomarkers to monitor subtle, early changes in LSEC function and the technical difficulties in isolating LSECs while preserving their physiological characteristics further hinder research efforts. In vitro models, while useful for studying specific aspects of LSEC function, lack the complexity of the in vivo environment [76], limiting their ability to capture the full spectrum of LSEC behavior.

### 8.2. Translational Obstacles

Translating findings is particularly challenging due to physiological differences between species. Rodent LSECs differ from human LSECs in key aspects, such as immune regulatory functions, making it difficult to model immune tolerance and disease progression accurately. Additionally, most existing models do not adequately replicate chronic liver conditions, such as MAFLD and cirrhosis, where long-term interactions between LSECs and other liver cells are crucial to disease progression. Addressing these challenges requires the development of more sophisticated models that better mimic human liver pathology.

## 9. Conclusions

In conclusion, LSECs play an essential role in regulating liver function, particularly in the context of immune responses and fibrosis in chronic liver diseases. Throughout this review, we have highlighted the unique anatomy and functions of LSECs, which allow them to act as critical modulators of hepatic homeostasis. LSECs not only filter blood and remove pathogens but also engage in immune regulation by presenting antigens and promoting immune tolerance. Their interaction with other hepatic cells, such as Kupffer cells, HSCs, and hepatocytes, is central to the maintenance of immune balance and prevention of excessive inflammation. However, when LSECs become dysfunctional, they contribute to the progression of liver diseases. Dysfunctional LSECs promote hepatic stellate cell activation and collagen deposition, leading to fibrosis, and exacerbating liver scarring, contributing to disease progression.

One of the key takeaways from this review is the understanding that LSECs are not merely passive endothelial cells but active regulators of the liver’s immune environment and fibrotic response. Changes in LSECs’ phenotype lead to dysregulated immune responses and fibrogenesis. This positions LSECs as both key drivers and potential therapeutic targets in chronic liver diseases. The ability of LSECs to modulate immune responses, interact with HSCs, and influence fibrosis progression underscores their significance in liver pathology.

Moving forward, LSECs represent a promising therapeutic target for both immune modulation and fibrosis prevention in liver disease. By targeting the pathways involved in LSEC dysfunction—such as nitric oxide production, TGF-β signaling, and capillarization—it may be possible to restore their normal function and prevent the progression of fibrosis. Furthermore, enhancing the regenerative potential of LSECs or harnessing their immune-regulatory capabilities could provide new strategies for treating chronic liver diseases and improving outcomes in liver transplantation. As research continues to unravel the complex roles of LSECs, their potential in therapeutic applications will likely play a pivotal role in shaping future treatments for liver diseases.

## Figures and Tables

**Table 1:** Summary of therapeutic approaches, emerging treatments, and future directions for targeting LSECs in liver fibrosis and immune modulation.

Therapeutic Approaches	Emerging Treatments	Drug Examples and Roles	Future Directions
Vasoactive Agents	Statins, Angiogenesis Inhibitors, FXR Agonists	Promote vascular stability and reduce fibrosis (e.g., Simvastatin).	Investigate how these treatments can restore LSEC function to improve overall hepatic microcirculation.
Shear Stress Modulators	Pirfenidone, Nintedanib, Galectin-3 Inhibitors	Modulate fibrosis progression (e.g., Nintedanib for antifibrotic effects).	Examine their ability to alleviate shear stress-induced damage to LSECs and enhance endothelial integrity.
TGF-β Signaling Inhibitors	Stem Cell Therapy, Gene Therapy	SB431542, LY2109761 to enhance stem cell engraftment and gene delivery.	Explore their role in creating an LSEC-supportive microenvironment for regenerative therapies.
Notch Pathway Modulators	LSEC-Targeted Nanoparticles, Controlled Release Systems	γ-secretase inhibitors (DAPT, MK-0752), Monoclonal Antibodies (OMP-59R5) to modulate LSEC dysfunction.	Investigate how targeting Notch signaling can reverse LSEC capillarization and promote vascular repair.
Immune Checkpoint Inhibitors	MicroRNA Modulators, Small Molecule Inhibitors	Nivolumab (anti-PD-1), Ipilimumab (anti-CTLA-4) to modulate immune responses and reduce liver inflammation.	Study their effects on reducing chronic inflammation and improving LSEC-mediated immune tolerance.
Cytokine Therapy	Combination Therapies (with emerging treatments like stem cell therapy)	Leverage cytokines to improve immune regulation in combination with other therapeutic approaches.	Investigate their potential in modulating LSEC-related inflammatory responses for better therapeutic outcomes.
